# “I am spiritual, but not religious”: Does one without the other protect against adolescent health-risk behaviour?

**DOI:** 10.1007/s00038-018-1116-4

**Published:** 2018-05-29

**Authors:** Klara Malinakova, Jaroslava Kopcakova, Andrea Madarasova Geckova, Jitse P. van Dijk, Jana Furstova, Michal Kalman, Peter Tavel, Sijmen A. Reijneveld

**Affiliations:** 1 OUSHI - Olomouc University Social Health Institute, Palacky University Olomouc, Univerzitni 244/22, 771 11 Olomouc, Czech Republic; 2 Department of Community and Occupational Health, University Medical Center Groningen, University of Groningen, Ant. Deusinglaan 1, 9713 AV Groningen, The Netherlands; 3 Graduate School Kosice Institute for Society and Health, University of Pavol Jozef Safarik Kosice, Tr. SNP 1, 040 11 Kosice, Slovak Republic; 4 Department of Health Psychology, Faculty of Medicine, University of Pavol Jozef Safarik Kosice, Tr. SNP 1, 040 11 Kosice, Slovak Republic; 5 Department of Social Medicine and Public Health, Faculty of Medicine and Dentistry, Palacky University Olomouc, Olomouc, Czech Republic; 6 Institute of Active Living, Faculty of Physical Culture, Palacky University Olomouc, Trida Miru 115, 771 11 Olomouc, Czech Republic

**Keywords:** Health-risk behaviour, Adolescence, Religious attendance, Spirituality, HBSC study

## Abstract

**Objectives:**

Spirituality and religious attendance (RA) have been suggested to protect against adolescent health-risk behaviour (HRB). The aim of this study was to explore the interrelatedness of these two concepts in a secular environment.

**Methods:**

A nationally representative sample (*n* = 4566, 14.4 ± 1.1 years, 48.8% boys) of adolescents participated in the 2014 Health Behaviour in School-aged Children cross-sectional study. RA, spirituality (modified version of the Spiritual Well-Being Scale), tobacco, alcohol, cannabis and drug use and the prevalence of sexual intercourse were measured.

**Results:**

RA and spirituality were associated with a lower chance of weekly smoking, with odds ratios (OR) 0.57 [95% confidence interval (CI) 0.36–0.88] for RA and 0.88 (0.80–0.97) for spirituality. Higher spirituality was also associated with a lower risk of weekly drinking [OR (95% CI) 0.91 (0.83–0.995)]. The multiplicative interaction of RA and spirituality was associated with less risky behaviour for four of five explored HRB. RA was not a significant mediator for the association of spirituality with HRB.

**Conclusions:**

Our findings suggest that high spirituality only protects adolescents from HRB if combined with RA.

**Electronic supplementary material:**

The online version of this article (10.1007/s00038-018-1116-4) contains supplementary material, which is available to authorized users.

## Introduction

Adolescent health-risk behaviour attracts the attention of researchers worldwide, because it can leave a lasting effect over the whole life course. The earlier onset of substance use, for example, is associated with engaging in multiple health-risk behaviours (Hansen et al. 2010) and is often a predictor of adult health-risk behaviour (Grant et al. 2006; Virtanen et al. 2015). Similarly, an early initiation of sexual life is associated with other risk factors (Lara and Abdo 2016).

With regard to prevalence, both gender differences (MacArthur et al. 2012; Saewyc et al. 1998; Wang et al. 2010) and country differences (Inchley et al. 2016) exist in adolescent health and health-risk behaviour. For example, in the 2005/2006 Health Behaviour in School-aged Children (HBSC) survey, the frequency of drunkenness increased by an average of 40% in all participating eastern European countries compared to the 1997/1998 HBSC survey, but decreased by an average of 25% in 13 of the 16 Western European and North American countries included in the study. An increasing trend in the Czech Republic, Bulgaria, Croatia and Hungary was reported also in the study of Kuntsche et al. (2011), which further pointed out that the prevalence remained stable or even decreased in countries such as Finland, Iceland and Norway. This finding shows the importance of the wider cultural and economical context and probably also reflects an effect of different policies in this area. From this perspective, the search for possible protective factors in adolescent health-risk behaviour remains an urgent need in the Czech Republic. According to the last published HBSC survey (2013/2014) (Inchley et al. 2016), the prevalence of drunkenness decreased significantly between the years 2010 and 2014. However, the Czech Republic still holds its position in the most unfavourable third of the countries with data on adolescent weekly drinking, in the unfavourable half regarding weekly smoking and recent cannabis use and in the least favourable ten per cent regarding early sexual intercourse.

Religiosity and spirituality have often been studied as protective factors in adolescent health-risk behaviour, including the prevention of smoking (Nonnemaker et al. 2006), alcohol (Piko et al. 2012) and cannabis use (Gmel et al. 2013) and sexual behaviour (Hardy and Raffaelli 2003; Nonnemaker et al. 2003). In a systematic review, Rew and Wong (2006) concluded that most studies (84%) showed that higher religiosity/spirituality was related to less health-damaging attitudes and behaviours. However, a minority of studies came to at least partially different conclusions. Burris et al. (2011) found religiosity to be associated with less underage alcohol use, while spirituality was associated with more, and also described a similar pattern regarding adolescent sexual behaviour (Burris et al. 2009).

The differences may be partly explained by the fact that both spirituality and religiosity are multidimensional constructs that include attitudes, behaviours and beliefs (Hooker et al. 2014). Nevertheless, many studies assess only one or two dimensions. Originally, the term religion included both individual and institutional dimensions (Hill and Pargament 2003); however, later it started to be more associated with religious institutions, prescribed theology and rituals and institutional beliefs and practices, such as church membership or attendance (Zinnbauer et al. 1997). In contrast, spirituality was originally used to describe a deeply religious attitude; however, recently, it is often also understood as a more subjective search for peace, harmony, meaning in life and connection with the sacred (Koenig 2008). The above-mentioned heterogeneity hinders comparison of the various studies. Though both religiousness and spirituality emphasize a search for the sacred, people who are religious or spiritual might differ in the means they use to find this. In the absence of religious commitment, an individual could actually even use alcohol, tobacco, hallucinogens or sexual intercourse, etc., as means to discover meaning, purpose and connectedness with the self, others or the transcendent (Burris et al. 2011).

However, other explanations may also hold for the varying associations of religiosity and spirituality. One of them is the degree of internalization of religious attitudes (Powell et al. 2003), i.e., the inner content and experience of one’s faith. This aligns with the spirituality level; therefore, it may be informative not only to analyse spirituality and religiosity separately, but also jointly, and to check a possible mediation effect. For the purpose of this article, we chose religious attendance as the external dimension of religiosity, and spirituality as the internal dimension. In our study, spirituality is understood in the broader sense: as the internal individual contentedness, one’s perceived closeness to God, one’s sense of meaning of life and of spiritual well-being (Ellison 1983).

Thus far, most studies on the relationship between religiosity/spirituality and adolescent health-risk behaviour have been conducted outside of Europe (Nonnemaker et al. 2006; Rew and Wong 2006), and only a very few within Central Europe (Brassai et al. 2015; Piko et al. 2012; Pitel et al. 2012). With regard to religious affiliation, the Czech Republic is a specific case in Central Europe. This might be the consequence of the historical development of the country, as the anticlerical attitudes that were already present were further reinforced by the 40 years of the communist régime (Nesporova and Nespor 2009). According to the Pew Research Center (2014), it is the country with the highest percentage (76.4%) of religiously unaffiliated people in the world, meaning that three-quarters of the population do not affiliate themselves to any organized church, though they might have some kind of personal belief. This very specific setting may affect the protective role of religiosity and spirituality regarding both physical and mental health (Hayward and Elliott 2014).

Therefore, the aim of this study is to explore the association of spirituality and religious attendance with adolescent health-risk behaviour in a highly secular environment and to explore whether spirituality modified the association of religious attendance, or religious attendance mediated that of spirituality.

## Methods

### Participants and procedure

We obtained data on a nationally representative sample of Czech boys and girls from the 2014 HBSC study. This cross-sectional WHO collaborative study focuses on health and health-related behaviour and their socio-economic determinants in 11-, 13-, and 15-year-old children. More detailed information about the survey can be found in Roberts et al. (2009). Schools were selected randomly after stratification by region, school size and type of school (primary schools vs. secondary schools). Out of 243 contacted schools, 242 agreed to participate (response rate 99.6%). Then, classes from the fifth, seventh and ninth grades, in general corresponding to age categories of 11-, 13- and 15-year-olds, were selected at random, one from each grade per school.

**Fig. 1:**
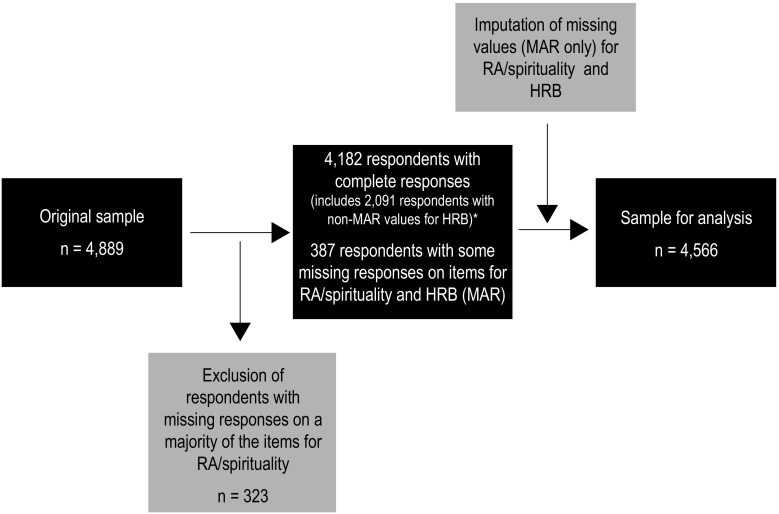
Preparation of the sample (Czech Republic, 2014). *Note:* *Items included only for the 15-year-old respondents; *RA* religious attendance, *MAR* values missing at random, *HRB* health-risk behaviour

Data from 14,539 pupils were obtained (response rate 89.2%). Most non-response was due to illness or other reasons, for example, sports or academic competitions (10.6%), and 30 children refused to participate in the survey (0.2%). The spirituality questionnaire was included only in the surveys of half of the 13- and 15-year-old adolescents, so the dataset comprised 4889 adolescents. Of these, 564 (11.5% of the sample) had not responded to at least one of the seven SWBS items. We used a multiple imputation to estimate values for the respondents who had responded to the majority of the SWBS items. The remaining participants—who had not responded to four or more SWBS items—were excluded from the study (*n* = 323). The final analytic sample thus included 4566 respondents (mean age = 14.4, SD = 1.09, 48.8% boys). For a graphical illustration of the preparation of the sample, see Fig. 1.

Data were collected between April and June 2014. The questionnaires were distributed by trained administrators with no teachers present in the classroom in order to reduce information bias. The consent to carry out the study was obtained through school management at all the schools involved in the survey. Participation in the survey was anonymous and voluntary, and the parents of the pupils were informed about the survey.

### Measures

*Religious attendance* was measured by the question: “How often do you go to church or to religious sessions?” with possible answers: several times a week/approximately once a week/approximately once a month/a few times a year/exceptionally/never. Sunday attendance is a matter of obligation in most of the Christian churches/denominations; therefore, the participants who reported attending religious sessions at least once a week were dichotomized as *attending*.

*Spirituality* was measured using the modified shortened version of the Spiritual Well-Being Scale (SWBS) (Cotton et al. 2005; Malinakova et al. 2017), measuring the overall spiritual well-being. Response possibilities for all seven items regarded a 6-point scale that ranged from “strongly disagree” (1) to “strongly agree” (6), leading to scores from 7 to 42. A higher score represented greater spiritual well-being. In the analyses, spirituality was used as a continuous variable, but for the purpose of dichotomization for a sensitivity analysis, participants with a score of 34 or higher (upper quartile of the score) were considered as spiritual, and the rest as non-spiritual. Cronbach’s alpha was 0.81 in our sample.

*Tobacco use* was measured by the question: “How often do you smoke tobacco at present?” Respondents reported their experience with smoking as follows: (1) every day; (2) at least once a week, but not every day; (3) less than once a week; (4) I do not smoke. Following the HBSC dichotomization (Currie et al. 2012), respondents who smoked at least once a week were classified as smokers, the rest as non-smokers.

*Alcohol use* was assessed by the question: “At present, how often do you drink anything alcoholic, such as beer, wine or spirits?” Respondents reported frequency of alcohol consumption for five types of alcohol drinks with the answers: (1) every day; (2) every week; (3) every month; (4) rarely; (5) never. Following the HBSC dichotomization (Currie et al. 2012), individuals were classified as alcohol consumers if they reported consumption of any alcohol drink at least each week.

*Cannabis use* was assessed only in the 15-year-old respondents. They were asked the question: “Have you taken cannabis (grass) in the last 30 days?” with the possible answers (1) never; (2) 1–2 days; (3) 3–5 days; (4) 6–9 days; (5) 10–19 days; (6) 20–29 days; (7) 30 days (and more). Following the HBSC dichotomization (Currie et al. 2012), respondents who answered “never” were classified as cannabis non-users, the rest of the respondents as users.

*Experience with drug use* was measured on 15-year-old respondents with the question “Have you ever taken one or several of these drugs in your life?” Respondents reported their lifetime experience with five kinds of drugs (ecstasy, pervitin, glue or solvents, LSD and a non-existing drug, netalin), with the same answers and dichotomization as for cannabis use. The respondents who reported an experience with netalin were not included in the analyses of lifetime drug use.

*Early sexual intercourse* was measured only among 15-year-old respondents by the question: “Have you ever had sexual intercourse (sometimes this is called “making love”, “having sex”, etc.)? (yes, no).

Age, gender and socio-economic status were considered as potential confounding variables. The socio-economic status of the respondents’ families was used as a covariate and was assessed by the Family Affluence Scale (FAS) (Currie et al. 2014). The scale examines the number of cars owned by the family, having one’s own bedroom, number of computers in the household, number of foreign family holidays, number of bathrooms and dishwasher ownership. The summary score ranges from 10 to 13, and following HBSC recommendations, it was converted into a fractional rank (ridit) score, leading to transformation of ordinal data to an interval scale with a normalized range (from 0 to 1, with higher score indicating higher socio-economic position) and distribution.

### Statistical analyses

As a first step, we performed a multiple imputation of missing data on item level, twenty times. It was assumed that data are missing at random (MAR). Then, we described the background characteristics of the sample and compared the respondents excluded from the analyses with the remaining ones. Next, we checked the effect of “school”, given the nested nature of the data. That showed that the intraclass correlation between students from the same school was negligible; therefore, we did not use multilevel modelling. We assessed the associations of only religious attendance (Model 1), only spirituality (Model 2), of both variables jointly (Model 3) and their multiplicative interaction (to assess moderation) (Model 4) with the various health-risk behaviours using binary logistic regression models. Each model was first tested as a crude one, and then, it was adjusted for gender, age and socio-economic status. For the sensitivity analysis using the dichotomized spirituality, the prevalences of all types of health-risk behaviour were compared with the proportion test. Finally, mediation analysis was performed using the bootstrap approach via *mediation* package in R. We tested whether religious attendance mediated the association of spirituality with health-risk behaviour as well as whether spirituality mediated the association of religious attendance with health-risk behaviour. All analyses were performed using the statistical software package IBM SPSS version 21. For the imputation of missing data, the *Hmisc* package in the R software was used.

## Results

The background characteristics of the sample are presented in Table 1, which also describe prevalence of five kinds of health-risk behaviour for both attending and non-attending respondents. Of the 4566 adolescents, 331 (7.2%) reported attending church services once a week or more. Religious attendance and spirituality (SWBS scale) were moderately correlated with Spearman’s *r* = 0.30 (*p *< 0.01). The mean SWBS score was 22.15 (SD = 7.61) with minimum 7 and maximum 42 (median 21). The SWBS was non-normally distributed, with skewness of 0.528 (SE = 0.036) and kurtosis of 0.063 (SE = 0.072). Of the highly spiritual respondents, i.e., those in the upper quartile of a score, 54.0% were boys and mean age was 14.31 (SD = 1.12). Of these, 61.9% were attending religious sessions at least once a week. Of the participants, 1202 (26.3%) were involved in at least one kind of health-risk behaviour, with the frequency being higher for non-attending (26.8%) than for attending (19.9%) respondents (*p *< 0.05). Compared to included respondents, those excluded (*n* = 323) were prevalently boys (*p *< 0.05), were slightly older (*p *< 0.01) and had a higher prevalence of recent cannabis (*p *< 0.05) and drugs use (*p *< 0.001), but did not differ significantly in regard to other health-risk behaviours.

**Table 1:** Characteristics of the sample (Czech Republic, 2014)

	Total		Religious attendance			
			Attending (≥ 1/week)		Non-attending (< 1/week)	
	Number	%	Number	%	Number	%
Gender						
Boys	2230	48.8	145	43.8	2085	49.2
Girls	2336	51.2	186	56.2	2150	50.8
Age						
13 years old (seventh grade)	2291	50.2	162	48.9	2129	50.3
15 years old (ninth grade)	2275	49.8	169	51.1	2106	49.7
Health-risk behaviour^a^						
Weekly smoking	487	10.7	23	6.9	464	11.0
Weekly drinking	577	12.6	33	10.0	544	12.8
Recent cannabis use (only 15-year-olds)	189	8.3	15	8.9	174	8.3
Lifetime drugs use (only 15-year-olds)	186	8.3	18	10.9	168	8.0
Early sexual intercourse (only 15-year-olds)	500	22.0	29	17.2	471	22.4
Total	4566	100	331	7.2	4235	92.8

^a^Only numbers regarding the respondents with the occurrence of a health-risk behaviour are presented

Table 2 shows the associations of religious attendance, spirituality and their interaction with various health-risk behaviours, adjusted for gender and age. Attending respondents were less likely to be involved only in weekly smoking, and the other associations were not statistically significant (Model 1). Similarly, a one SD increase in spirituality was associated with a 12% decrease in the odds of weekly smoking and a 9% decrease in the odds of weekly drinking (Model 2). When religious attendance and spirituality were both added to the model (Model 3), neither of them was statistically significant for any type of health-risk behaviour. The interaction of religious attendance and spirituality (Model 4) showed that a one SD increase in spirituality for attending respondents was associated with 40% decrease in the odds of weekly smoking, 31% decrease in the odds of weekly drinking, 51% decrease in the odds of recent cannabis use and 52% decrease in the odds of lifetime drug use. With regard to early sexual intercourse, the result was significant only for the crude model (33% decrease in the odds), but not for the adjusted one.

**Table 2:** Associations of adolescent weekly smoking, weekly drinking, recent cannabis use, lifetime drugs use and early sexual intercourse with religious attendance, spirituality (standardized to *z*-scores), their joint association and their interaction, adjusted for age, gender and socio-economic status (FAS) (odds ratios, OR, and 95% confidence intervals, CI) (Czech Republic, 2014)

	Weekly smoking		Weekly drinking		Recent cannabis use (15 years old)		Lifetime drugs use (15 years old)		Early sexual intercourse (15 years old)	
	Crude	Adjusted	Crude	Adjusted	Crude	Adjusted	Crude	Adjusted	Crude	Adjusted
Model 1: religious attendance										
Non-attending	**1 (ref)**	**1 (ref)**	**1 (ref)**	**1 (ref)**	**1 (ref)**	**1 (ref)**	**1 (ref)**	**1 (ref)**	**1 (ref)**	**1 (ref)**
Attending	0.61 (0.39–0.94)*	0.57 (0.36–0.88)*	0.75 (0.52**–**1.09)	0.74 (0.51**–**1.08)	1.08 (0.62**–**1.88)	1.03 (0.59**–**1.80)	1.40 (0.84**–**2.34)	1.39 (0.83**–**2.32)	0.72 (0.48**–**1.09)	0.67 (0.44**–**1.01)
Model 2: spirituality (per SD)	0.84 (0.76–0.92)***	0.88 (0.80–0.97)*	0.91 (0.83–0.996)*	0.91 (0.83–0.995)*	0.93 (0.80**–**1.09)	0.91 (0.78**–**1.07)	1.05 (0.90**–**1.22)	1.06 (0.91**–**1.24)	0.98 (0.88**–**1.08)	0.95 (0.85**–**1.05)
Model 3: religious attendance and spirituality mutually adjusted^a^										
Attending versus non-attending	0.75 (0.48**–**1.18)	0.64 (0.40**–**1.02)	0.84 (0.57**–**1.24)	0.82 (0.55–1.23)	1.22 (0.67–2.23)	1.19 (0.65–2.16)	1.39 (0.78–2.45)	1.35 (0.76–2.38)	0.71 (0.46–1.10)	0.68 (0.44–1.07)
Spirituality (per SD)	**0.86 (0.77**–0.95)**	0.91 (0.82–1.01)	0.93 (0.84–1.02)	0.92 (0.84–1.02)	0.91 (0.77–1.08)	0.90 (0.76–1.07)	1.007 (0.85–1.19)	1.02 (0.86–1.21)	1.01 (0.90–1.13)	0.98 (0.88–1.10)
Model 4: interaction^b^										
Attendance versus non-attendance	1.10 (0.68–1.80)	0.96 (0.58–1.60)	1.18 (0.75–1.87)	1.17 (0.73–1.88)	**2.00 (1.08**–3.72)*	**1.88 (1.01**–3.53)*	**2.54 (1.34**–4.82)**	**2.54 (1.34**–4.83)**	1.04 (0.61–1.78)	0.96 (0.56–1.66)
Spirituality (per SD)	**0.89 (0.80**–0.995)*	0.95 (0.85–1.06)	0.96 (0.87–1.06)	0.96 (0.86–1.06)	1.01 (0.84–1.20)	0.99 (0.82–1.18)	1.10 (0.92–1.31)	1.12 (0.94–1.34)	1.05 (0.93–1.18)	1.02 (0.90–1.14)
Religious attendance × spirituality (per SD)	**0.61 (0.43**–0.87)**	**0.60 (0.41**–0.87)**	**0.69 (0.50**–0.95)*	**0.69 (0.50**–0.95)*	**0.47 (0.29**–0.78)**	**0.49 (0.30**–0.82)**	**0.50 (0.30**–0.82)**	**0.48 (0.29**–0.80)**	**0.67 (0.46**–0.98)*	0.70 (0.47–1.02)

*p < 0.05; **p < 0.01; ***p < 0.001; Those with* p*-values below 0.05 are considered significant and are shown in bold; *SD* standard deviation

^a^Model 3: logit(Health-risk behaviour) = *α* + *β*_1_ * RA + *β*_2_ * spirituality + *β*_3_ * gender + *β*_4_ * age + *β*_5_ * SES + *ε*

^b^Model 4: logit(Health-risk behaviour) = *α* + *β*_1_ * RA + *β*_2_ * spirituality + *β*_3_ * RA * spirituality + *β*_4_ * gender + *β*_5_ * age + *β*_6_ * SES + *ε*

**Fig. 2:**
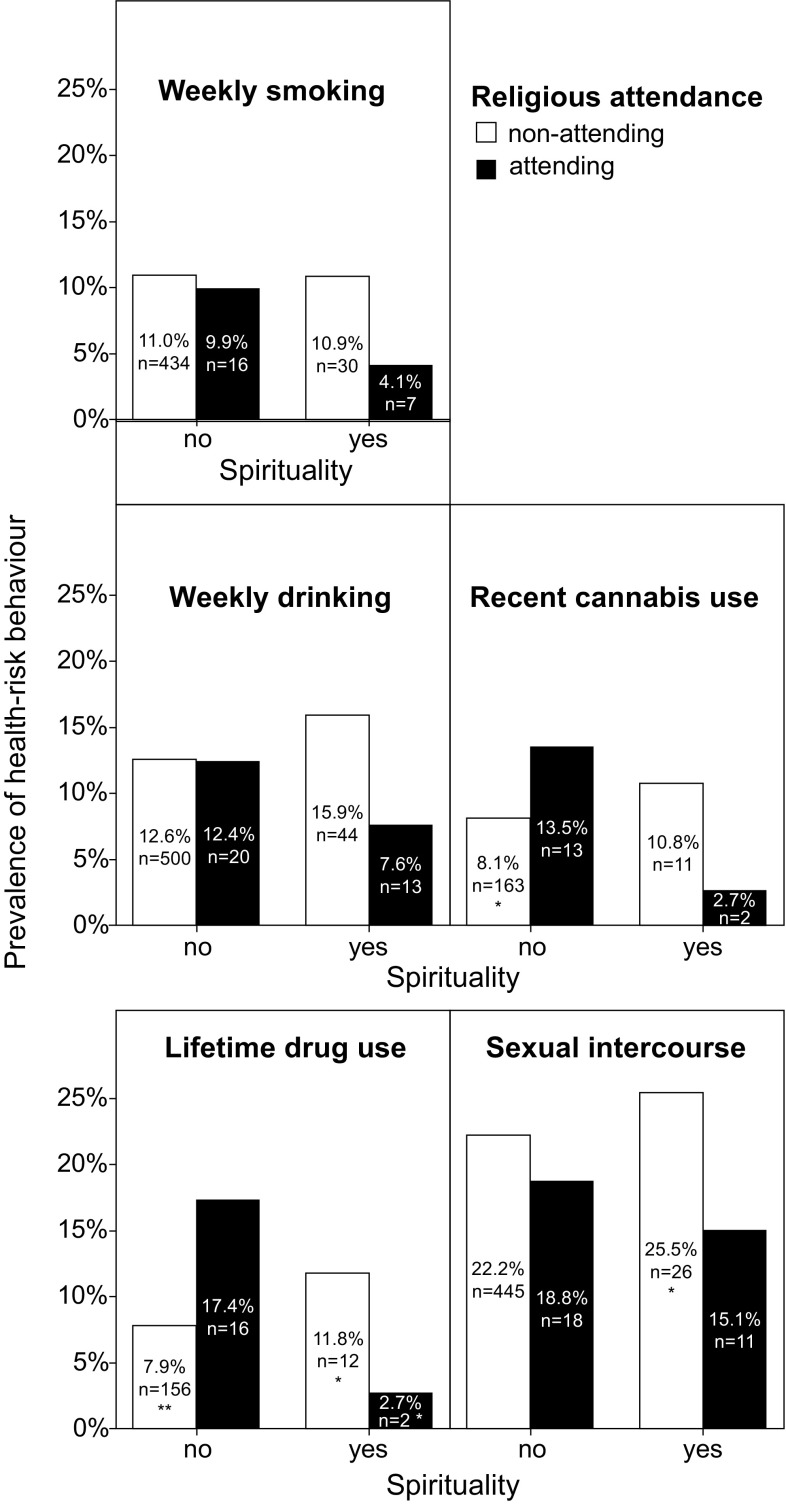
Prevalence of adolescent weekly smoking, weekly drinking, recent cannabis use, lifetime drugs use and early sexual intercourse in groups with different combinations of spirituality and religious attendance; *p < 0.05; **p < 0.001 (Czech Republic, 2014)

The sensitivity analysis using the dichotomized spirituality (Fig. 2) compared the prevalences of health-risk behaviour in respective groups with the proportion test. Non-spiritual attending group (NSA) was considered reference group for these comparisons in order to allow a more detailed assessment of the dissonance of religious attendance and spirituality. *p < 0.05, **p < 0.001.

Comparison of prevalences of health-risk behaviour (Fig. 2) showed that there were no significant differences in the prevalence of smoking and weekly drinking in the respective groups. The recent cannabis use had significantly higher prevalence in the NSA (13.5, 95% CI 9.6–17.4%) than the non-spiritual non-attending group (NSNA) (8.1, 7.2–9.0%). The lifetime drug use had significantly higher prevalence in the NSA (17.4, 13.1–21.7%) than all other groups: the NSNA (7.9, 7.0–8.8%), the spiritual non-attending group (11.8, 8.6–15.0%), and the spiritual attending group (2.7, 0.0–8.5%). On the other hand, the prevalence of sexual intercourse in the NSA group was significantly lower (18.8, 14.3–23.3%) than in the NSNA (25.5, 21.2–29.8%).

Religious attendance was not a significant mediator for the association of spirituality with health-risk behaviour (*p *> 0.10 for all types of health-risk behaviour). On the other hand, spirituality was a significant mediator for the association of religious attendance with smoking only (*p *= 0.03); it was not a significant mediator for religious attendance with other types of health-risk behaviour (*p *> 0.10 for all types of health-risk behaviour except for smoking).

## Discussion

The aim of this study was to assess the relationship of religious attendance, spirituality and their interaction with health-risk behaviour among adolescents in a highly secular environment. The results showed that mere religious attendance and spirituality were associated with only one or two kinds of health-risk behaviour, but their multiplicative interaction was associated with four of the five behaviours examined. Attending respondents and spiritual respondents were less likely to be regular smokers, and spiritual adolescents were less likely to overuse alcohol. The associations were not significant for cannabis, drug use and early sexual intercourse. We also found that religious attendance and spirituality were not associated with health-risk behaviour in case of mutual adjustment. Moreover, with the exception of smoking, the religious attendance and spirituality were not mediators for each other for the association with health-risk behaviour.

The association of religious attendance and spirituality with less risk behaviour as we found in our study is consistent with previous findings of other authors (Kub and Solari-Twadell 2013; Rew and Wong 2006). Religious attendance and spirituality may influence risk behaviour via several pathways. First, religious systems generally emphasize one’s responsibility to care for health and discourage behaviours that could harm the body (Koenig 2012). Second, parents of religious respondents show a stronger parental monitoring of adolescents’ behaviour (Mahoney 2010), which may to a certain degree prevent the occurrence of unwanted behaviours. Third, religious organizations offer different leisure-time activities, which may also serve as a prevention of some risk behaviours (Adamczyk and Felson 2012). It requires further analysis which would include also the additional variables to discriminate between these explanations.

However, we also found that the interaction of a low level of spirituality and religious attendance was associated with an increased level of health-damaging behaviours, which differs from the findings of Pitel et al. (2012). This study dealt with a similar issue in Slovak adolescents, but found the religious/non-spiritual group not to be so distinct from the other groups as we found. An explanation could be the different cultural contexts of Slovakia and the Czech Republic—religiosity is distinctly more prevalent in Slovakia (85.3% Christian) than in the Czech Republic (23.3% Christian) (Pew Research Center 2014). A second explanation may be the different way of assessing spirituality, i.e., using a question on the importance of faith by Pitel et al. (2012) versus using the spirituality questionnaire as we did, with the latter probably being a stronger measure.

Our finding of a higher prevalence of some risk behaviours among adolescents who attend but are not spiritual raises important questions about this specific group, which has rarely been studied. Some adolescents may attend church services without an adequate internal conviction. We could argue that their religious practice is more the result of external pressure, usually from the family. Thus, the experienced discrepancy could result in a desire to rebel in some way, for example, by health-damaging behaviour. In addition, this discrepancy may lead to substantial existential distress, causing individuals to regulate their emotions in maladaptive ways, for example, through alcohol or drug use (Aldwin et al. 2014). At the same time, higher spirituality was associated with less likely weekly smoking and drinking, but not with the other risk behaviours. Therefore, the popular being “spiritual, but not religious” might have only a limited impact on someone’s behaviour, as some other authors also concluded (Jang and Franzen 2013).

### Strengths and limitations

This study has several important strengths, the most important being its large and representative sample and its high response rate. It is also the first study that uses the shortened version of the SWBS in the Czech environment. However, the high proportion of non-attending respondents (92.8%) and the correspondingly low number of attending respondents represent a limitation of our study, as it decreased the power of the study in particular regarding moderation. Another limitation might be information bias, as our data were based on self-reports of adolescents, which can be influenced by social desirability. A third limitation is the cross-sectional design of the study which does not allow us to make conclusions on causality.

### Implications

Our findings suggest that taking care of the spiritual and religious needs of adolescents may affect their risk behaviours. Such care could include, for example, family and school education as well as pastoral care focussing on promoting the process of finding one’s own identity and the healthy spirituality of the adolescent. We found that, in particular, religious attendance without strong spirituality may not be protective or can even increase the likelihood of health-risk behaviour. This could lead to educating parents on the deleterious effects of forcing adolescents to attend church without internal spiritual drive. Alternatively, our results support the idea that the more effective interventions would be the ones that lead to internalization of the spiritual values. During adolescence, relationships with their peers represent a strong factor influencing the adolescents’ behaviour and attitudes. Therefore, a useful strategy to prevent adolescent health-risk behaviour might be to create an environment where spiritual values are shared and respected by the whole group, for example, in scout and other organizations, or different activities in youth centres.

Our results also show that the available evidence on religiosity and spirituality should be interpreted with caution. It is important to keep in mind the multidimensionality of both constructs and the consequent ambiguity in definitions and methods of measurement. A group of “religious respondents” may include participants with different levels of spirituality, which could lead to misinterpretation of results. Future research on this topic and on the causal pathway is therefore recommended.

### Conclusion

Our findings suggest that religious attendance or spirituality separately have only limited impact on adolescent health-risk behaviour. Spirituality may only protect against health-risk behaviour if combined with religious attendance, and if not the reverse holds true for attendance without being spiritual. Thus, this study shows the importance of the internalization of adolescent religious values with and its impact on health-risk behaviour, inviting for more attention for research on this theme.

## Electronic supplementary material

Below is the link to the electronic supplementary material.

Supplementary material 1 (DOCX 31 kb)
