# Supplementary material for: “I am spiritual, but not religious”: Does one without the other protect against adolescent health-risk behaviour?
Source: Int J Public Health. 2018 May 29;64(1):115–24. doi: 10.1007/s00038-018-1116-4 (PMC6353810; doi:10.1007/s00038-018-1116-4)
Supplement: Supplementary file 1 — Supplementary material 1 (DOCX 31 kb) [file 38_2018_1116_MOESM1_ESM.docx]

**International Journal of Public health:** **“I am spiritual, but not religious.” Does one without the other protect against adolescent** **health-risk behaviour?**

**The shortened version of the Spiritual Well-Being Scale (1)**

In the survey, the questionnaire was presented as follows:

**We are going to present you ten statements. For each statement, mark the number that most expresses your consent or disagreement with your personal experience.**

*Read every statement carefully. Please, notice, to which degree you agree with it.*

|  | Strongly disagree  1   | 2   | 3   | 4   | 5   | Strongly agree  6   |
| --- | --- | --- | --- | --- | --- | --- |
| 1. ~~I don’t know who I am, where I came from, or where I’m going.~~ | 🞎 | 🞎 | 🞎 | 🞎 | 🞎 | 🞎 |
| 1. I believe that God loves me and cares about me. | 🞎 | 🞎 | 🞎 | 🞎 | 🞎 | 🞎 |
| 1. I have a personally meaningful relationship with God. | 🞎 | 🞎 | 🞎 | 🞎 | 🞎 | 🞎 |
| 1. I feel very fulfilled and satisfied with my life. | 🞎 | 🞎 | 🞎 | 🞎 | 🞎 | 🞎 |
| 1. ~~I don’t get much personal strength and support from God.~~ | 🞎 | 🞎 | 🞎 | 🞎 | 🞎 | 🞎 |
| 1. I believe that God is concerned about my problems. | 🞎 | 🞎 | 🞎 | 🞎 | 🞎 | 🞎 |
| 1. I feel good about my future. | 🞎 | 🞎 | 🞎 | 🞎 | 🞎 | 🞎 |
| 1. ~~Life doesn’t have much meaning.~~ | 🞎 | 🞎 | 🞎 | 🞎 | 🞎 | 🞎 |
| 1. My relationship with God contributes to my sense of well-being | 🞎 | 🞎 | 🞎 | 🞎 | 🞎 | 🞎 |
| 1. I believe there is some real purpose for my life. | 🞎 | 🞎 | 🞎 | 🞎 | 🞎 | 🞎 |

Based on a psychometric analysis of the scale (2), the negatively worded items (a, e, h) were excluded and only the modified seven-item version of the scale was used.

**References:**

1. Cotton S, Larkin E, Hoopes A, Cromer BA, Rosenthal SL. The impact of adolescent spirituality and religiosity on depressive symptoms and health risk behaviour. J Adolesc Health. 2005;36:529.e7-.e14.

2. Malinakova K, Kopcakova J, Kolarcik P, Geckova AM, Solcova IP, Husek V, et al. The Spiritual Well-Being Scale: Psychometric Evaluation of the Shortened Version in Czech Adolescents. Journal of religion and health. 2017;56(2):697-705.
